# 2-Chloro-9-isopropyl-*N*,*N*-dimethyl-9*H*-purin-6-amine

**DOI:** 10.1107/S1600536810011797

**Published:** 2010-04-02

**Authors:** Michal Rouchal, Marek Nečas, Robert Vícha

**Affiliations:** a Department of Chemistry, Faculty of Technology, Tomas Bata University in Zlin, Nám. T. G. Masaryka 275, Zlín,762 72, Czech Republic; b Department of Chemistry, Faculty of Science, Masaryk University in Brno, Kamenice 5, Brno-Bohunice, 625 00, Czech Republic

## Abstract

In the title compound, C_10_H_14_ClN_5_, the imidazole and pyrimidine rings are essentially planar [maximum deviation = 0.0013 (14) and 0.0207 (13) Å, respectively]. In the crystal, the mol­ecules are linked by weak C—H⋯N inter­actions into chains parallel to the *c* axis and the crystal packing is stabilized by additional weak C—H⋯N and C—H⋯Cl inter­actions.

## Related literature

The title compound was prepared according to a modification of the procedure of Fiorini & Abel (1998 ▶). For the synthesis and/or biological activity of related compounds, see: Legraverend & Grierson (2006 ▶). For related structures, see: Kubicki & Codding (2001 ▶); Trávníček & Popa (2007 ▶); Rouchal *et al.* (2009*a* ▶,*b* ▶,*c* ▶).
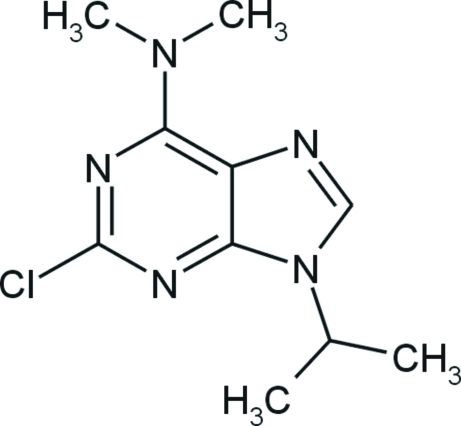


## Experimental

### 

#### Crystal data

C_10_H_14_ClN_5_
*M* *_r_* = 239.71
Monoclinic, 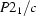

*a* = 12.0483 (3) Å
*b* = 8.7689 (2) Å
*c* = 11.5538 (3) Å
β = 109.965 (3)°
*V* = 1147.30 (5) Å^3^
*Z* = 4
Mo *K*α radiation
μ = 0.31 mm^−1^
*T* = 120 K
0.40 × 0.40 × 0.30 mm

#### Data collection

Oxford Diffraction Xcalibur (Sapphire2 large Be window) diffractometer
Absorption correction: multi-scan (*CrysAlis RED*; Oxford Diffraction, 2009 ▶) *T*_min_ = 0.968, *T*_max_ = 1.000
13393 measured reflections
2022 independent reflections
1798 reflections with *I* > 2σ(*I*)
*R*_int_ = 0.016

#### Refinement

*R*[*F* ^2^ > 2σ(*F* ^2^)] = 0.025
*wR*(*F* ^2^) = 0.068
*S* = 1.05
2022 reflections
149 parameters
H-atom parameters constrained
Δρ_max_ = 0.21 e Å^−3^
Δρ_min_ = −0.18 e Å^−3^

### 

Data collection: *CrysAlis CCD* (Oxford Diffraction, 2009 ▶); cell refinement: *CrysAlis RED* (Oxford Diffraction, 2009 ▶); data reduction: *CrysAlis RED*; program(s) used to solve structure: *SHELXS97* (Sheldrick, 2008 ▶); program(s) used to refine structure: *SHELXL97* (Sheldrick, 2008 ▶); molecular graphics: *ORTEP-3* (Farrugia, 1997 ▶) and *Mercury* (Macrae *et al.*, 2008 ▶); software used to prepare material for publication: *SHELXL97*.

## Supplementary Material

Crystal structure: contains datablocks global, I. DOI: 10.1107/S1600536810011797/pk2238sup1.cif

Structure factors: contains datablocks I. DOI: 10.1107/S1600536810011797/pk2238Isup2.hkl

Additional supplementary materials:  crystallographic information; 3D view; checkCIF report

## Figures and Tables

**Table 1:** Hydrogen-bond geometry (Å, °)

*D*—H⋯*A*	*D*—H	H⋯*A*	*D*⋯*A*	*D*—H⋯*A*
C4—H4*A*⋯N1^i^	0.95	2.49	3.3728 (18)	154
C7—H7*C*⋯Cl1^ii^	0.98	2.91	3.5981 (14)	128
C7—H7*B*⋯N3^iii^	0.98	2.75	3.584 (2)	143
C9—H9*A*⋯N3^iv^	0.98	2.73	3.6664 (18)	161

Symmetry codes: (i) 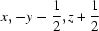; (ii) 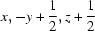; (iii) 
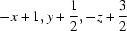
; (iv) 
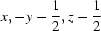
.

**Fig. 1.**
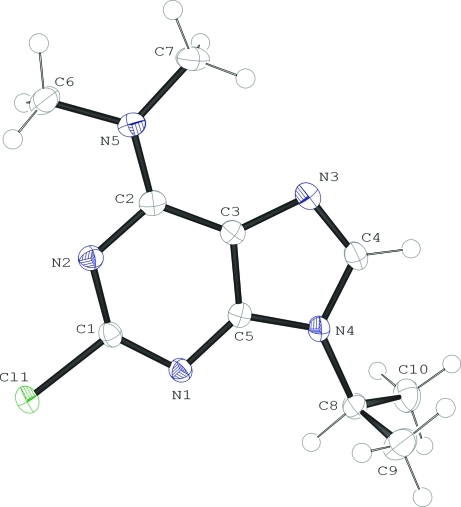
An ellipsoid plot (50% probability) of the asymmetric unit. Hydrogen atoms are represented as arbitrary spheres.

**Fig. 2.**
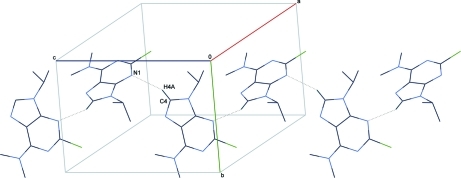
A view of the crystal structure showing chains parallel to the a-axis linked via C—H···N contacts (dotted lines). H-atoms (except those which are involved in H-bonding) have been omitted for clarity.

## supplementary crystallographic information

### Comment

The heterocyclic system, imidazo[4,5-*d*]pyrimidine, commonly known as purine, was first named by Emil Fisher at the turn of the 19^th^ century. A large number of variously substituted purines exhibit a wide range of biological activities (Legraverend & Grierson, 2006). They act as interferon inducers, adenosine receptor ligands, inhibitors of microtubule assembly, protein kinases, sulfotransferases and phosphodiesterases.  The title molecule was prepared as a part of our research into the synthesis of novel trisubstituted purines.

The asymmetric unit of the title compound consists of a single purine molecule.  Both imidazole and pyrimidine rings are nearly planar with maximum deviations from the mean plane being 0.0013 (14) Å for C4 (imidazole ring) and 0.0207 (13) Å for C2 (pyrimidine ring).  Both carbon atoms of the dimethylamino substituent lie essentially in the pyrimidine mean plane as demonstrated by torsion angles C3—C2—N5—C7 and C3—C2—N5—C6, which are 4.3 (2)° and 175.90 (13)°, respectively.  The torsion angle describing the orientation of isopropyl and purine ring, H8A—C8—N4—C4 is -163.55 (13)°. Molecules are linked into chains along the *c* axis by weak C4—H4···N1 interactions (Table 1, Fig. 2). Crystal packing is further stabilised by short C—H···N and C—H···Cl contacts (Table 1).

### Experimental

The title compound was prepared according to a slightly modified literature procedure (Fiorini & Abel, 1998). 2,6-Dichloro-9-(propan-2-yl)-9*H*-purine (0.87 mmol, 196 mg) and methylamine hydrochloride (0.91 mmol, 61.5 mg) were dissolved in a mixture of DMF (2.5 ml) and *N*-ethyl-*N*-isopropylpropan-2-amine (1.74 mmol, 225 mg). The resulting solution was stirred at 90 °C for 2 hours. Subsequently, the mixture was diluted with water and extracted with diethyl ether. Combined organic layers were washed twice with brine and dried over Na_2_SO_4_. Crude product consisting of two compounds with relative abundances of 43% and 57% according to GC were obtained after evaporation of the solvent in vacuum. The products were identified as *N*-methyl and *N*,*N*-dimethyl derivatives.  Column chromatography (silica gel; petroleum ether/ethyl acetate, v/v, 1/1) yielded the latter as a colourless crystalline powder (105 mg, 54%, mp 418–422 K). The crystal used for data collection was grown by spontaneous evaporation from deuterochloroform at room temperature.

### Crystal data

**Table:**

C_10_H_14_ClN_5_	*F*(000) = 504
*M**_r_* = 239.71	*D*_x_ = 1.388 Mg m^−^^3^
Monoclinic, *P*2_1_/*c*	Melting point = 422–418 K
Hall symbol: -P 2ybc	Mo *K*α radiation, λ = 0.7107 Å
*a* = 12.0483 (3) Å	Cell parameters from 8720 reflections
*b* = 8.7689 (2) Å	θ = 2.9–27.3°
*c* = 11.5538 (3) Å	µ = 0.31 mm^−^^1^
β = 109.965 (3)°	*T* = 120 K
*V* = 1147.30 (5) Å^3^	Block, colourless
*Z* = 4	0.40 × 0.40 × 0.30 mm

### Data collection

**Table:**

Oxford Diffraction Xcalibur (Sapphire2 large Be window) diffractometer	2022 independent reflections
Radiation source: Enhance (Mo) X-ray Source	1798 reflections with *I* > 2σ(*I*)
graphite	*R*_int_ = 0.016
Detector resolution: 8.4 pixels mm^-1^	θ_max_ = 25.0°, θ_min_ = 2.9°
ω scans	*h* = −14→13
Absorption correction: multi-scan (*CrysAlis RED*; Oxford Diffraction, 2009)	*k* = −9→10
*T*_min_ = 0.968, *T*_max_ = 1.000	*l* = −13→13
13393 measured reflections	

### Refinement

**Table:**

Refinement on *F*^2^	Primary atom site location: structure-invariant direct methods
Least-squares matrix: full	Secondary atom site location: difference Fourier map
*R*[*F*^2^ > 2σ(*F*^2^)] = 0.025	Hydrogen site location: inferred from neighbouring sites
*wR*(*F*^2^) = 0.068	H-atom parameters constrained
*S* = 1.05	*w* = 1/[σ^2^(*F*_o_^2^) + (0.0355*P*)^2^ + 0.4207*P*] where *P* = (*F*_o_^2^ + 2*F*_c_^2^)/3
2022 reflections	(Δ/σ)_max_ < 0.001
149 parameters	Δρ_max_ = 0.21 e Å^−^^3^
0 restraints	Δρ_min_ = −0.18 e Å^−^^3^

### Special details

**Table:**

Experimental. CrysAlis RED (Oxford Diffraction Ltd, 2009). Empirical absorption correction using spherical harmonics, implemented in SCALE3 ABSPACK scaling algorithm.
Geometry. All esds (except the esd in the dihedral angle between two l.s. planes) are estimated using the full covariance matrix. The cell esds are taken into account individually in the estimation of esds in distances, angles and torsion angles; correlations between esds in cell parameters are only used when they are defined by crystal symmetry. An approximate (isotropic) treatment of cell esds is used for estimating esds involving l.s. planes.
Refinement. Refinement of *F*^2^ against ALL reflections. The weighted *R*-factor wR and goodness of fit *S* are based on *F*^2^, conventional *R*-factors *R* are based on *F*, with *F* set to zero for negative *F*^2^. The threshold expression of *F*^2^ > σ(*F*^2^) is used only for calculating *R*-factors(gt) etc. and is not relevant to the choice of reflections for refinement. *R*-factors based on *F*^2^ are statistically about twice as large as those based on *F*, and *R*- factors based on ALL data will be even larger.

### Fractional atomic coordinates and isotropic or equivalent isotropic displacement parameters (Å^2^)

**Table:**

	*x*	*y*	*z*	*U*_iso_*/*U*_eq_	
Cl1	0.78721 (3)	0.18146 (4)	0.33908 (3)	0.02432 (12)	
N1	0.80758 (9)	−0.04481 (12)	0.48883 (9)	0.0166 (2)	
N2	0.67313 (9)	0.15775 (12)	0.49228 (10)	0.0179 (3)	
N3	0.69089 (10)	−0.14494 (13)	0.72589 (10)	0.0225 (3)	
N4	0.82037 (10)	−0.23853 (13)	0.64258 (10)	0.0184 (3)	
N5	0.55582 (10)	0.16106 (13)	0.61327 (10)	0.0197 (3)	
C1	0.75175 (11)	0.08580 (15)	0.45552 (11)	0.0168 (3)	
C2	0.63956 (11)	0.09009 (15)	0.58130 (11)	0.0169 (3)	
C3	0.69562 (11)	−0.04950 (15)	0.63134 (11)	0.0167 (3)	
C4	0.76612 (12)	−0.25408 (16)	0.72857 (13)	0.0229 (3)	
H4A	0.7816	−0.3366	0.7851	0.027*	
C5	0.77572 (11)	−0.10713 (15)	0.57988 (11)	0.0157 (3)	
C6	0.51096 (13)	0.30962 (16)	0.56040 (14)	0.0256 (3)	
H6A	0.4933	0.3068	0.4711	0.038*	
H6B	0.5707	0.3880	0.5968	0.038*	
H6C	0.4388	0.3337	0.5778	0.038*	
C7	0.51768 (12)	0.10497 (17)	0.71274 (13)	0.0248 (3)	
H7A	0.5191	−0.0068	0.7134	0.037*	
H7B	0.4372	0.1406	0.6998	0.037*	
H7C	0.5711	0.1435	0.7917	0.037*	
C8	0.90697 (12)	−0.34193 (16)	0.61883 (13)	0.0214 (3)	
H8A	0.9468	−0.2849	0.5691	0.026*	
C9	0.84438 (14)	−0.47859 (18)	0.54375 (14)	0.0314 (4)	
H9A	0.7866	−0.4433	0.4660	0.047*	
H9B	0.8038	−0.5358	0.5904	0.047*	
H9C	0.9024	−0.5447	0.5265	0.047*	
C10	1.00104 (13)	−0.38877 (18)	0.73954 (14)	0.0294 (3)	
H10A	1.0359	−0.2974	0.7868	0.044*	
H10B	1.0627	−0.4476	0.7221	0.044*	
H10C	0.9650	−0.4515	0.7874	0.044*	

### Atomic displacement parameters (Å^2^)

**Table:**

	*U*^11^	*U*^22^	*U*^33^	*U*^12^	*U*^13^	*U*^23^
Cl1	0.0311 (2)	0.0225 (2)	0.02385 (19)	0.00422 (14)	0.01520 (15)	0.00769 (13)
N1	0.0180 (5)	0.0170 (6)	0.0149 (5)	−0.0002 (4)	0.0058 (4)	0.0005 (4)
N2	0.0189 (6)	0.0173 (6)	0.0176 (5)	0.0002 (4)	0.0062 (4)	−0.0005 (4)
N3	0.0256 (6)	0.0227 (6)	0.0219 (6)	0.0007 (5)	0.0115 (5)	0.0038 (5)
N4	0.0198 (6)	0.0168 (6)	0.0189 (6)	0.0020 (4)	0.0071 (5)	0.0037 (5)
N5	0.0196 (6)	0.0200 (6)	0.0204 (6)	0.0021 (5)	0.0081 (5)	−0.0020 (5)
C1	0.0192 (7)	0.0170 (7)	0.0136 (6)	−0.0023 (5)	0.0046 (5)	−0.0003 (5)
C2	0.0162 (6)	0.0174 (7)	0.0152 (6)	−0.0031 (5)	0.0031 (5)	−0.0048 (5)
C3	0.0169 (6)	0.0174 (7)	0.0155 (6)	−0.0025 (5)	0.0049 (5)	−0.0019 (5)
C4	0.0267 (7)	0.0221 (8)	0.0219 (7)	0.0014 (6)	0.0109 (6)	0.0067 (6)
C5	0.0149 (6)	0.0153 (7)	0.0149 (6)	−0.0017 (5)	0.0025 (5)	−0.0016 (5)
C6	0.0264 (7)	0.0228 (8)	0.0280 (8)	0.0070 (6)	0.0097 (6)	−0.0013 (6)
C7	0.0231 (7)	0.0284 (8)	0.0276 (7)	−0.0006 (6)	0.0148 (6)	−0.0038 (6)
C8	0.0202 (7)	0.0212 (7)	0.0250 (7)	0.0051 (6)	0.0105 (6)	0.0055 (6)
C9	0.0347 (9)	0.0262 (8)	0.0329 (8)	0.0061 (7)	0.0110 (7)	−0.0036 (7)
C10	0.0225 (7)	0.0306 (8)	0.0326 (8)	0.0044 (6)	0.0064 (6)	0.0094 (7)

### Geometric parameters (Å, °)

**Table:**

Cl1—C1	1.7575 (13)	C6—H6A	0.9800
N1—C1	1.3174 (17)	C6—H6B	0.9800
N1—C5	1.3522 (17)	C6—H6C	0.9800
N2—C1	1.3230 (17)	C7—H7A	0.9800
N2—C2	1.3630 (17)	C7—H7B	0.9800
N3—C4	1.3112 (18)	C7—H7C	0.9800
N3—C3	1.3926 (17)	C8—C9	1.520 (2)
N4—C5	1.3696 (17)	C8—C10	1.5228 (19)
N4—C4	1.3698 (18)	C8—H8A	1.0000
N4—C8	1.4769 (17)	C9—H9A	0.9800
N5—C2	1.3402 (17)	C9—H9B	0.9800
N5—C7	1.4610 (18)	C9—H9C	0.9800
N5—C6	1.4618 (18)	C10—H10A	0.9800
C2—C3	1.4220 (19)	C10—H10B	0.9800
C3—C5	1.3900 (18)	C10—H10C	0.9800
C4—H4A	0.9500		
			
C1—N1—C5	109.04 (11)	N5—C6—H6C	109.5
C1—N2—C2	117.66 (11)	H6A—C6—H6C	109.5
C4—N3—C3	104.13 (11)	H6B—C6—H6C	109.5
C5—N4—C4	105.59 (11)	N5—C7—H7A	109.5
C5—N4—C8	126.37 (11)	N5—C7—H7B	109.5
C4—N4—C8	128.02 (11)	H7A—C7—H7B	109.5
C2—N5—C7	121.94 (11)	N5—C7—H7C	109.5
C2—N5—C6	120.29 (11)	H7A—C7—H7C	109.5
C7—N5—C6	117.26 (11)	H7B—C7—H7C	109.5
N1—C1—N2	132.14 (12)	N4—C8—C9	110.23 (11)
N1—C1—Cl1	113.90 (10)	N4—C8—C10	110.34 (11)
N2—C1—Cl1	113.95 (10)	C9—C8—C10	112.28 (12)
N5—C2—N2	116.82 (12)	N4—C8—H8A	107.9
N5—C2—C3	125.83 (12)	C9—C8—H8A	107.9
N2—C2—C3	117.35 (11)	C10—C8—H8A	107.9
C5—C3—N3	109.72 (11)	C8—C9—H9A	109.5
C5—C3—C2	116.22 (12)	C8—C9—H9B	109.5
N3—C3—C2	134.05 (12)	H9A—C9—H9B	109.5
N3—C4—N4	114.04 (12)	C8—C9—H9C	109.5
N3—C4—H4A	123.0	H9A—C9—H9C	109.5
N4—C4—H4A	123.0	H9B—C9—H9C	109.5
N1—C5—N4	125.94 (12)	C8—C10—H10A	109.5
N1—C5—C3	127.51 (12)	C8—C10—H10B	109.5
N4—C5—C3	106.52 (11)	H10A—C10—H10B	109.5
N5—C6—H6A	109.5	C8—C10—H10C	109.5
N5—C6—H6B	109.5	H10A—C10—H10C	109.5
H6A—C6—H6B	109.5	H10B—C10—H10C	109.5

### Hydrogen-bond geometry (Å, °)

**Table:**

*D*—H···*A*	*D*—H	H···*A*	*D*···*A*	*D*—H···*A*
C4—H4A···N1^i^	0.95	2.49	3.3728 (18)	154
C7—H7C···Cl1^ii^	0.98	2.91	3.5981 (14)	128
C7—H7B···N3^iii^	0.98	2.75	3.584 (2)	143
C9—H9A···N3^iv^	0.98	2.73	3.6664 (18)	161

Symmetry codes: (i) *x*, −*y*−1/2, *z*+1/2; (ii) *x*, −*y*+1/2, *z*+1/2; (iii) −*x*+1, *y*+1/2, −*z*+3/2; (iv) *x*, −*y*−1/2, *z*−1/2.
